# Intaglio Surface Accuracy of Temporary Crowns Fabricated by Additive Versus Subtractive CAD/CAM Methods

**DOI:** 10.3390/ma19194109

**Published:** 2026-09-25

**Authors:** Ana Paula Ayres, Nicole Rodrigues, Otavio Henrique Pinhata-Baptista, Guillermo Pradies, Arthur Rodriguez Gonzalez Cortes

**Affiliations:** 1 Department of Stomatology, School of Dentistry, University of São Paulo, São Paulo 05508-000, Brazil; ana.ayres.lima@gmail.com (A.P.A.); otaviopinhata@gmail.com (O.H.P.-B.); 2 Department of Conservative Dentistry and Prosthodontics, School of Dentistry, Complutense University of Madrid, 28040 Madrid, Spain; nicoleci@ucm.es (N.R.); gpradies@odon.ucm.es (G.P.); 3 Department of Dental Surgery, Faculty of Dental Surgery, University of Malta, MSD 2080 Msida, Malta

**Keywords:** temporary crowns, milling, 3D printing, internal fit, marginal fit, intaglio surface accuracy, additive manufacturing, CAD/CAM

## Abstract

The success of temporary restorations depends on their marginal and internal adaptation, which are influenced by the manufacturing method. This study evaluated the intaglio surface accuracy, internal fit, and marginal fit of provisional crowns fabricated via subtractive (milling) and additive (3D printing) CAD/CAM technologies. Three milling systems—CEREC Primemill (4-axis), PrograMill Dry (5-axis), and Zirkonzahn M1 (5-axis)—and three 3D printers—Phrozen Mini 4K (LCD), SprintRay Pro95S (DLP), and Stratasys Objet30 (PolyJet)—were evaluated. For 3D printing, five build orientations (0°, 30°, 45°, 60°, and 90°) were tested. A total of 360 crowns were fabricated. Accuracy was quantified by Root Mean Square (RMS) deviation, and fit was assessed using the triple-scan method. Results indicated that build angles of 0° and 30° yielded the best performance for printed crowns (*p* < 0.05). While milling systems showed significantly superior intaglio accuracy and marginal fit compared to 3D printing (*p* < 0.05), no significant differences were found in internal fit between the two methods (*p* > 0.05). Milling offers higher dimensional predictability, but both approaches are clinically viable when optimized parameters are utilized.

## 1. Introduction

Temporary restorations are indispensable components of reconstructive dental treatments, serving critical roles in pulp protection, maintenance of periodontal health, occlusal stability, and aesthetic diagnosis [1,2,3,4]. To fulfill these roles effectively, these restorations must exhibit characteristics comparable to definitive prostheses, particularly regarding marginal adaptation and internal fit [5,6]. These parameters are decisive factors for the clinical seating of the crown and the prevention of microleakage, which can lead to secondary caries and endodontic failure [7,8].

The consolidation of digital workflows in dentistry has significantly transformed the fabrication of provisional crowns through Computer-Aided Design and Computer-Aided Manufacturing (CAD/CAM) systems [9,10]. This shift has enabled greater control and standardization by replacing traditional manual steps with automated processes [11,12]. Digitalization reduces the reliance on individual technical skill, offering higher predictability and the ability to design complex geometries that align with the mechanical properties of modern resins [13,14,15].

Within this digital framework, manufacturing can be executed via subtractive methods, such as the milling of pre-polymerized polymethyl methacrylate (PMMA) blocks, or additive methods, like the three-dimensional (3D) printing of photopolymerizable resins [16]. Milling is recognized for its high dimensional stability and precision due to the use of industrial blocks with a high degree of conversion [17,18]. However, it is also associated with significant material waste and limitations when detailing highly complex internal geometries due to the diameter of the milling burs [19,20].

Conversely, 3D printing offers a versatile alternative, allowing for the creation of intricate shapes with minimal material consumption [21,22]. Despite these advantages, the accuracy of additive manufacturing is highly sensitive to numerous variables, including the specific printing technology—such as Digital Light Processing (DLP), Liquid Crystal Display (LCD), or Photopolymer Jetting (PolyJet)—as well as operational parameters like layer thickness and build orientation [23,24,25]. The current lack of standardized protocols and comparative evidence between these diverse systems contributes to heterogeneous results in the literature [26,27].

Based on these findings, we hypothesized that the intaglio surface accuracy of temporary crowns would be significantly affected by the CAM method and parameters. Thus, the objective of the present study was to evaluate the intaglio surface accuracy, marginal adaptation, and internal fit of provisional crowns fabricated using both additive and subtractive CAD/CAM methods. Specifically, the study aimed to compare different milling configurations (4-axis and 5-axis) against various 3D printing technologies (DLP, LCD, and PolyJet). Furthermore, the investigation sought to determine the influence of five different build orientations (0°, 30°, 45°, 60°, and 90°) on the overall accuracy and adaptation of the printed restorations.

## 2. Materials and Methods

A prefabricated PMMA tooth, representing a maxillary first molar (tooth 26) with a double-chamfer finish line preparation, was used as the master model. Digital acquisition was performed using a high-precision intraoral scanner (Primescan 1, CEREC 5, Dentsply Sirona, Bensheim, Germany). This scanner utilizes parallel confocal microscopy to capture high-resolution optical images at varying focal depths, which are then processed for precise 3D reconstruction.

The scanned STL file was imported into Exocad software (DentalCAD 3.1, Exocad GmbH, Darmstadt, Germany) for the design of the provisional crown. The design parameters were strictly standardized: the internal cement space was set at 80 μm, beginning 1 mm above the cervical finish line. In the marginal region, a space of 20 μm was programmed. Four reference spheres (1 mm diameter) were added to the center of the buccal, palatal, mesial, and distal surfaces to ensure standardized cross-sectional positioning during the fit evaluation.

**Figure 1:**
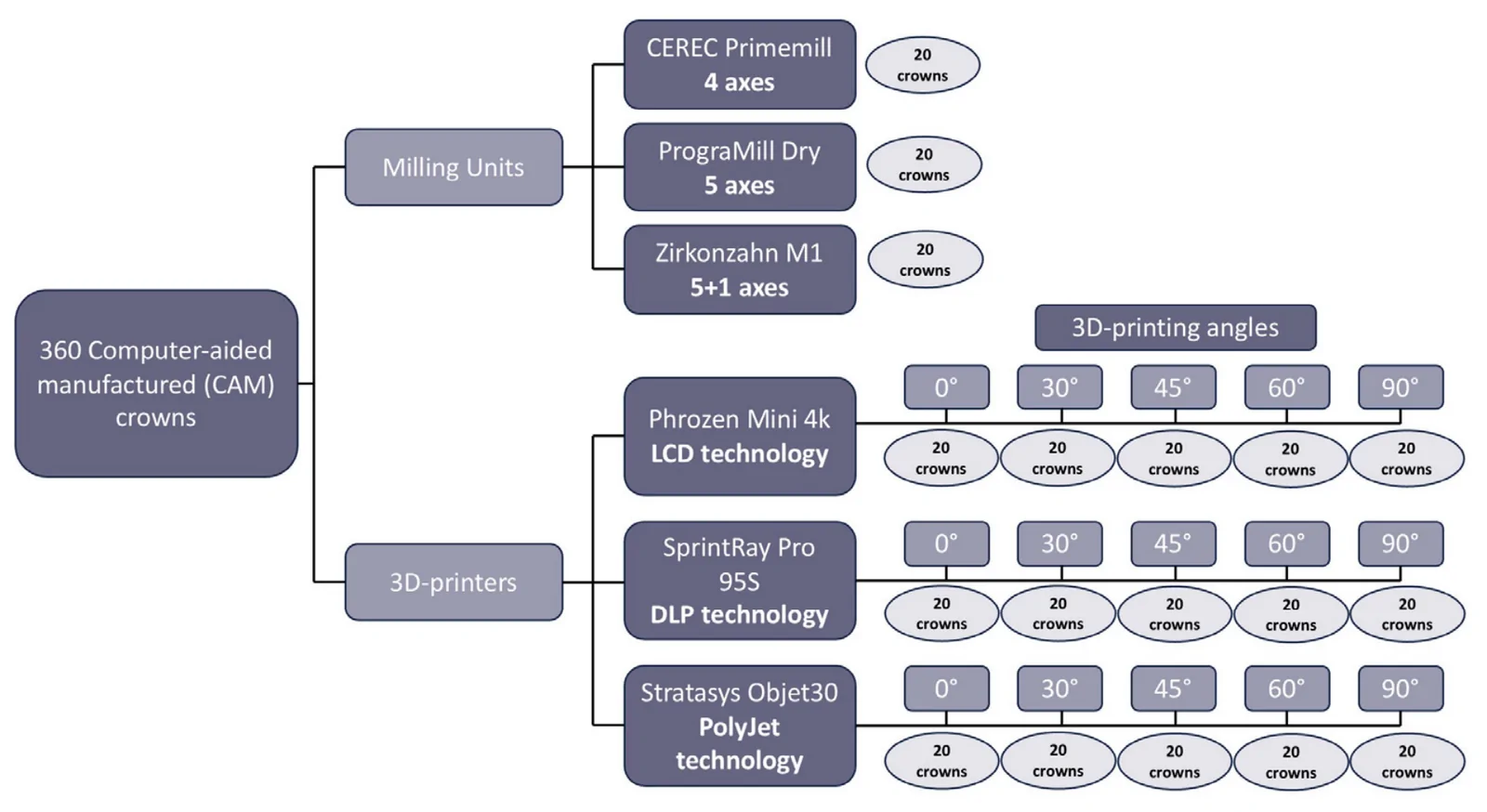
Flowchart of the study.

A total of 360 crowns were fabricated and randomly divided into two groups according to the manufacturing method (Figure 1). The first group (n = 60) comprised three subtractive manufacturing systems (i.e., milling units) with different numbers of axes: CEREC Primemill Dentsply Sirona, Bensheim, Germany) (4-axis unit; n = 20), PrograMill Dry (Dry cycle, Ivoclar Vivadent, Schaan, Liechtenstein), (5-axis unit; n = 20), and M1 (Zirkonzahn, Gais, Italy) (5+1-axis unit; n = 20). In the Zirkonzahn M1, the additional axis (+1) is auxiliary and used only for the automatic changing of blanks and tools, with no direct involvement in the machining process. The second group (n = 300) comprised three additive manufacturing systems (i.e., 3D printers) based on different technologies: Phrozen Mini 4K (Phrozen Tech Co., Ltd., Hsinchu, Taiwan) (LCD technology; n = 100), SprintRay Pro95S (SprintRay Inc., Los Angeles, CA, USA) (DLP technology; n = 100), and Stratasys Objet30 (Stratasys, Eden Prairie, MN, USA) (PolyJet technology; n = 100). For each printer, the 100 specimens were further divided into five subgroups of 20 crowns according to the build orientation angles: 0°, 30°, 45°, 60°, and 90°.

**Figure 2:**
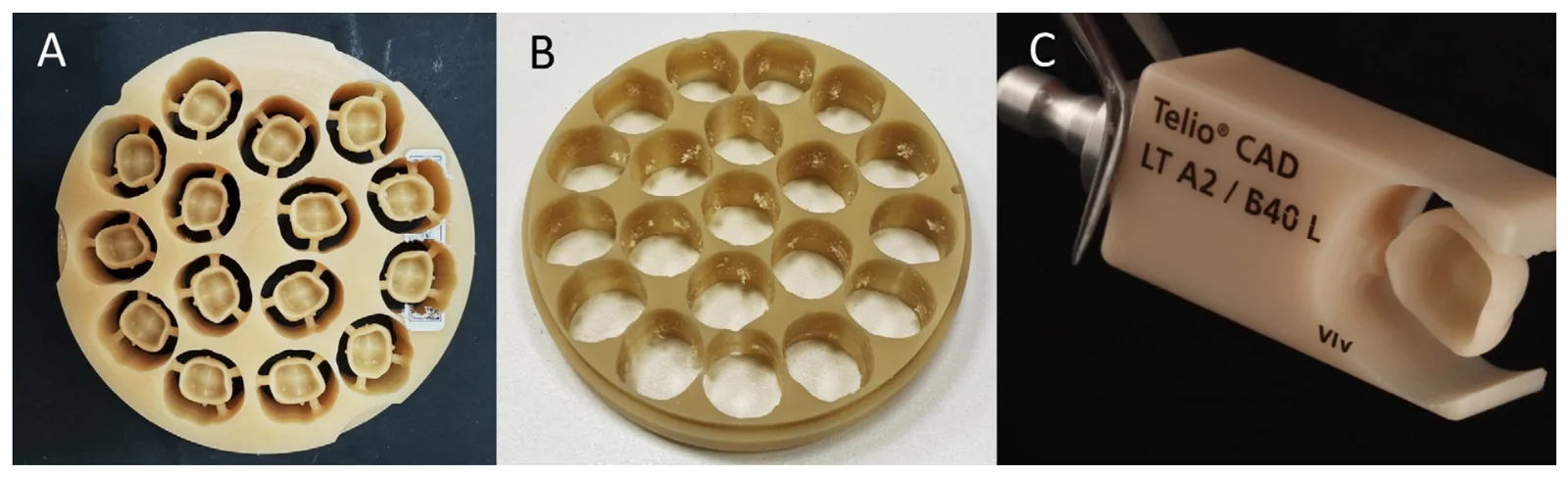
Milled PMMA materials tested: Temp Basic A3 Blank (Zirkonzahn GmbH), milled with a Zirkonzahn M1 (**A**); Huge PMMA Block Monolayer A3 disk (Shandong Huge Dental Material Co.), milled with a PrograMill Dry (**B**); and Telio CAD LT A2 block (Ivoclar Vivadent), milled with a CEREC Primemill (**C**).

The CEREC Primemill (Dentsply Sirona, Bensheim, Germany) is a 4-axis milling unit optimized for processing Telio CAD LT A2 blocks (14 × 12 × 18 mm) using an “extra-fine” milling protocol. Its toolset includes a 1.0 mm PMMA CS ball-end bur for fine finishing in narrow areas, alongside 1.2 mm and 1.4 mm diamond ball-end burs specifically designed for anatomical refinement and the definition of fissures and larger internal geometries. For bulk material removal and external milling, the system utilizes a 2.5 mm flat-end cylindrical bur. In contrast, the PrograMill Dry (Ivoclar Vivadent) utilizes a 5-axis configuration to process Huge PMMA Monolayer A3 discs (98 mm diameter, 14 mm thickness) in “high definition” mode. This system employs a specialized 0.7 mm ball-end tool for ultra-fine finishing of narrow regions and fissures, a standard 1.0 mm ball-end tool for general anatomical contours, and a 2.5 mm flat-end cylindrical tool for the initial roughing stage and external milling. Finally, the Zirkonzahn M1 features a 5+1 axis movement range, processing 95 mm Temp Basic A3 discs under a “high definition” setting. Its milling sequence relies on a 1.0 mm short flat-end cylindrical bur for precise marginal finishing, a 1.5 mm ball-end bur for internal and anatomical refinement, and a 2.0 mm flat-end cylindrical bur for the primary reduction in the material block. All systems tested utilized industrial PMMA blocks and specific burs according to the manufacturer’s recommendations (Figure 2).

**Figure 3:**
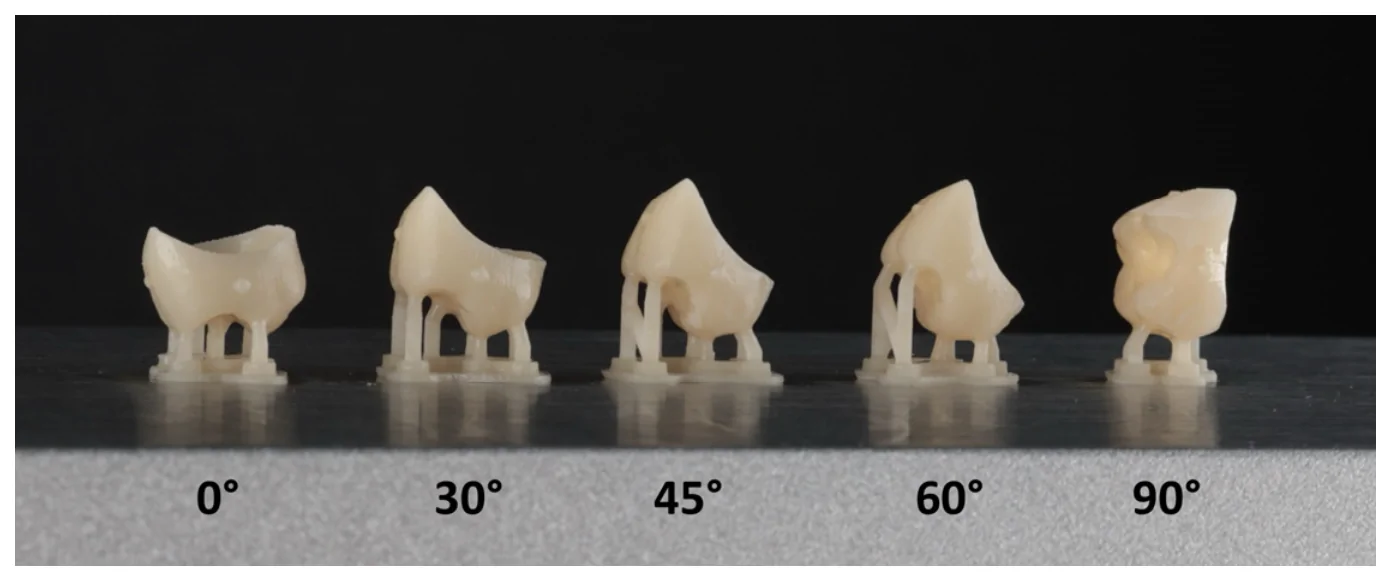
Three-dimensional-printing building orientation angles tested in the study.

The 3D printers tested were calibrated prior to the study using standard test objects to verify dimensional accuracy. For all angles tested, post-processing followed manufacturer-specific protocols (Figure 3). For LCD and DLP systems, crowns were washed in 99.9% isopropyl alcohol (IPA). The PolyJet system used an automated support removal process.

Several factors of additive manufacturing protocols for dental restorations vary significantly across LCD, DLP, and PolyJet technologies for the devices tested. The Phrozen Mini 4K (LCD) utilizes VarseoSmile Temp resin at a 50 μm layer thickness, controlled via Chitubox Free (v.1.2.1) software. This system offers high granularity in editable parameters, including complex exposure transitions (linear or exponential), light-off delays, and specific lift/retraction kinematics for base and regular layers. Post-processing involves a 5-min non-heated ultrasonic IPA bath followed by a 10-min UV cure at 405 nm.

Similarly, the SprintRay Pro 95S (DLP—SprintRay Inc., Los Angeles, CA, USA) processes the same VarseoSmile Temp resin at a 50 μm resolution through RayWare software (v2.9.2.5), though its parameters focus more on crown orientation and support geometry. Its post-processing is highly automated, utilizing the Pro Wash/Dry system for an 11-min cycle and a rapid 1 min and 50-s cure in the ProCure unit.

In contrast, the Objet 30 Dental Prime utilizes PolyJet technology to jet VeroGlaze MED620 photopolymer and SUP705 support material at a much finer layer thickness of 16 μm. Managed by Objet Studio (v2_7_5984), the primary editable variable is the print angle. Unlike the resin-vat systems, PolyJet requires no external post-curing unit as polymerization occurs during the printing process; however, it necessitates a pressurized WaterJet system to mechanically remove support material.

All temporary crowns produced in the study were scanned within 24 h of fabrication using the Primescan intraoral scanner (CEREC 5, Dentsply Sirona, Bensheim, Germany). This timeframe was established to obtain high-fidelity virtual models of the specimens and to mitigate potential dimensional distortions associated with prolonged storage. All scanning procedures were conducted by a single operator, who performed equipment calibration prior to the commencement of each experimental group. To minimize variables related to operator fatigue, a five-minute rest interval was implemented between the scanning of different groups.

**Figure 4:**
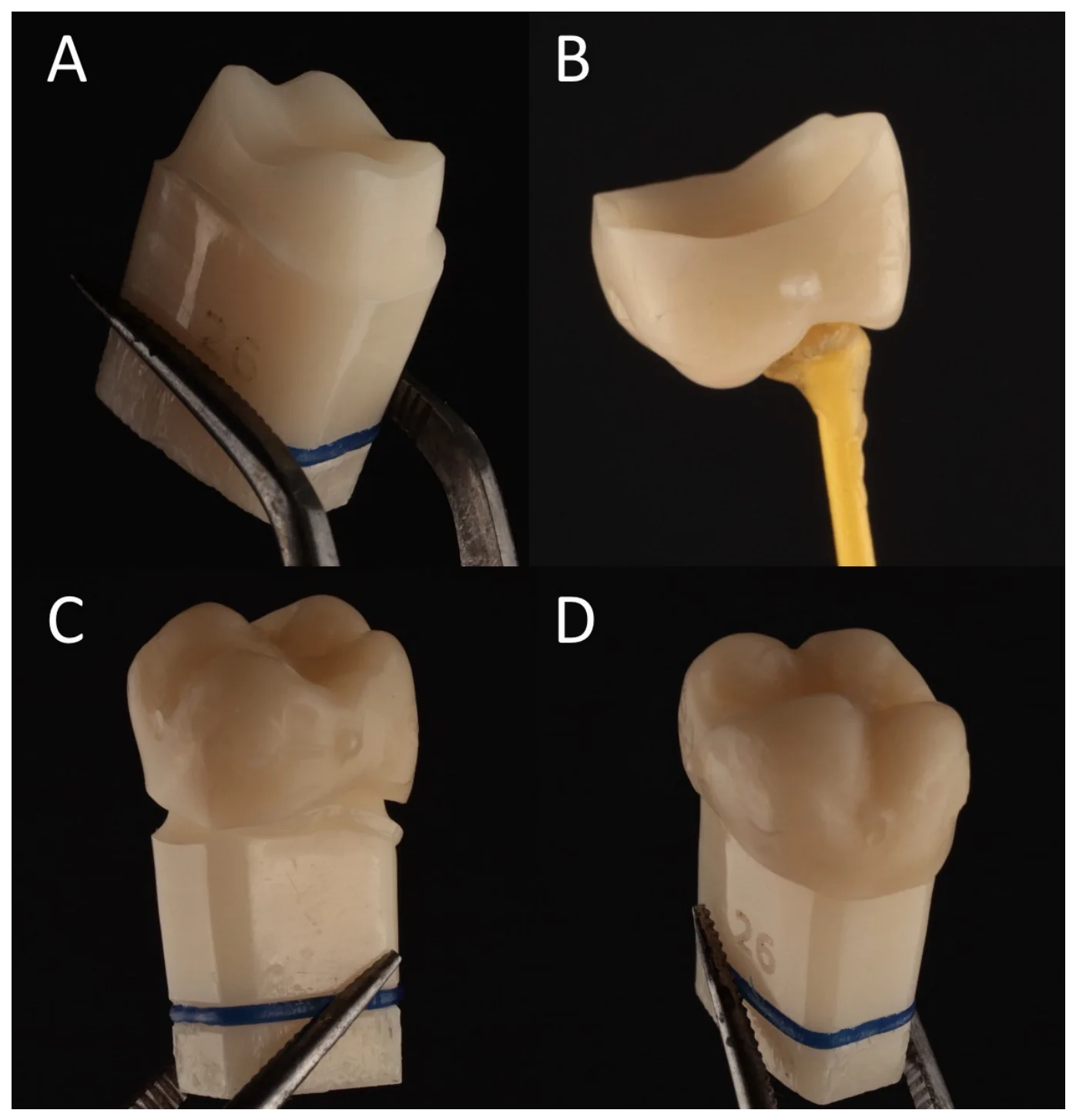
Scanning stages of the fabricated provisional crowns: (**A**) scanning of the tooth preparation; (**B**) scanning of the provisional crown; (**C**) seating of the provisional crown on the tooth preparation; and (**D**) scanning of the provisional crown in its final seating position on the tooth preparation.

During the procedure, the crowns were secured using adhesive-tip applicators (OptraStick, Ivoclar Vivadent, Schaan, Liechtenstein) positioned on the occlusal surface, facilitating the initial digitization of the intaglio (internal) surface. Subsequently, the applicators were repositioned and fixed to the internal surface to allow for the scanning of the occlusal aspect. This comprehensive protocol ensured the full acquisition of both the external and internal geometries of each sample (Figure 4).

To evaluate the internal surface accuracy of the manufactured provisional crowns, the STL files of the scanned crowns and the reference CAD files were imported into the three-dimensional analysis software Geomagic Control X (v2025, 3D Systems, Rock Hill, SC, USA). The analysis was conducted by a single operator with specific training in Geomagic Control X and previous experience applying this analytical workflow in research studies.

Within the software environment, the digital meshes were aligned using the Iterative Closest Point (ICP) algorithm via the global best-fit alignment method. This algorithm establishes correspondences between the point clouds of the two meshes and executes successive iterations until the distance between the compared objects is minimized. The best-fit alignment was performed considering the entire restoration. By avoiding forced alignment in specific regions, a more representative measurement of the overall accuracy of the specimen was achieved.

Following the superposition of the meshes, the intaglio surface of the crowns was selected. From this selection, a 3D comparison analysis was generated, featuring a color-coded map visualization and the extraction of numerical data, including minimum, maximum, and mean values, mean positive and negative deviations, variance, and the Root Mean Square (RMS) value. The RMS was utilized as the primary metric to quantify the internal surface accuracy of each crown, representing the quadratic mean of point-to-point deviations between the scanned model and the CAD reference. Low RMS values indicate high three-dimensional congruence between the compared surfaces.

The formula used for the calculation of the RMS was:

RMS = √(1/n ∑_i=1_^n^ (x_1,i_ − x_2,i_)^2^) (1)

where

- x_1,i_ is the coordinate of point i in the reference CAD model;
- x_2,i_ is the corresponding coordinate in the scan of the fabricated crown;
- n is the total number of points evaluated.

**Figure 5:**
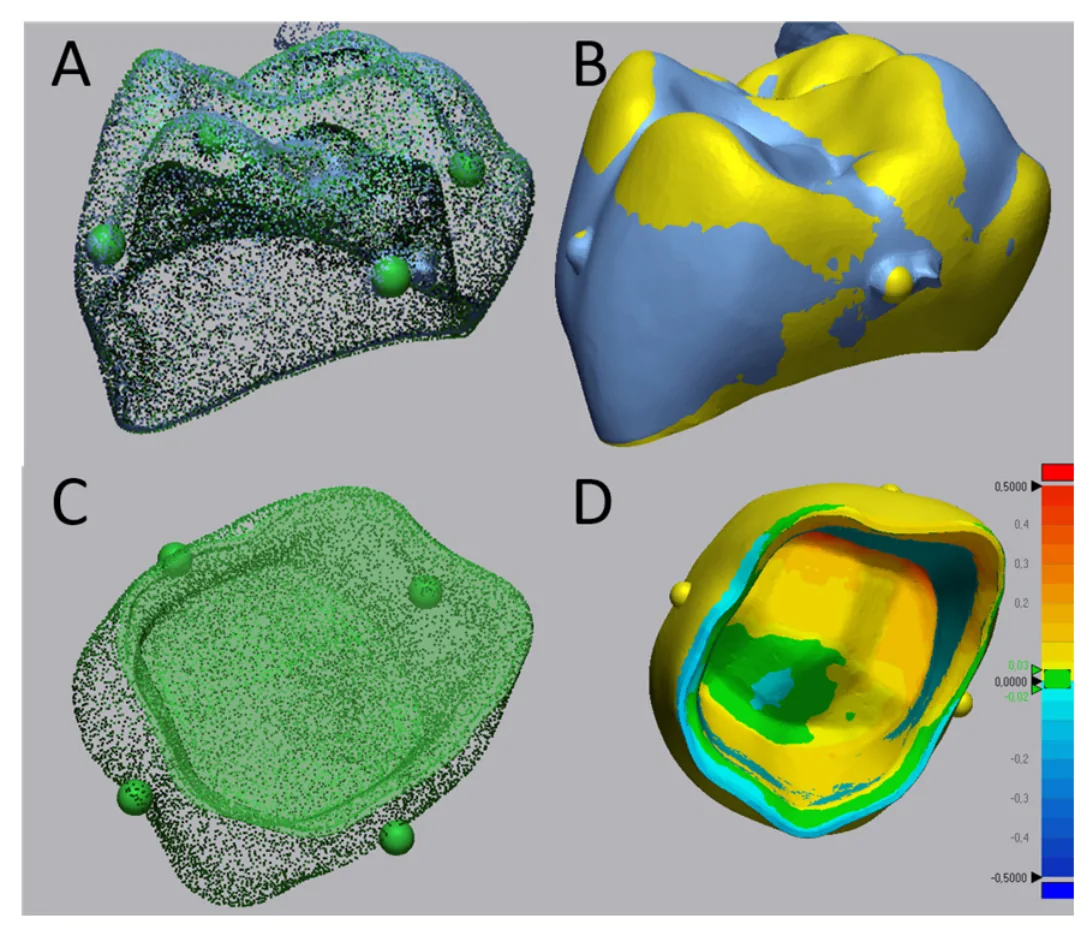
Stages of the internal surface accuracy evaluation for provisional crowns: selection of all points from the CAD file and the STL file of the scanned crown (**A**), best-fit alignment of the CAD file with the STL file of the scanned crown (**B**), selection of the crown intaglio surface (**C**), and calculation of deviations between the CAD and the scanned crown on the selected internal surface, with color-map visualization (**D**).

The entire intaglio surface of the crown was included in the analysis, encompassing both the internal area and the marginal region (Figure 5). Internal surface accuracy was defined according to the ISO 5725-1:2023 standard as a parameter composed of two complementary components: trueness and precision. Trueness was represented by the mean of the RMS values, indicating the degree of conformity between the fabricated specimen and the digital reference model. Precision was represented by the standard deviation of the RMS values, reflecting the consistency of the results within each experimental group.

Marginal and internal fit were assessed via the triple-scan method in Zeiss Inspect software (v2025) (Carl Zeiss GOM Metrology GmbH, Braunschweig, Germany). All triple-scan measurements were performed by the same operator, who had been specifically trained in the Zeiss Inspect workflow and had previously employed this methodology in other research studies. Cross-sections were executed based on 0.5 mm reference spheres previously positioned at the center of the buccal, palatal, mesial, and distal surfaces of each specimen. From these reference points, two sectioning planes were established: one in the mesiodistal direction and the other in the buccopalatal direction. These sections provided two-dimensional visualizations of the interface between the crown and the tooth preparation, allowing for linear measurements of the distances between the internal surfaces of the restoration and the external surfaces of the preparation.

**Figure 6:**
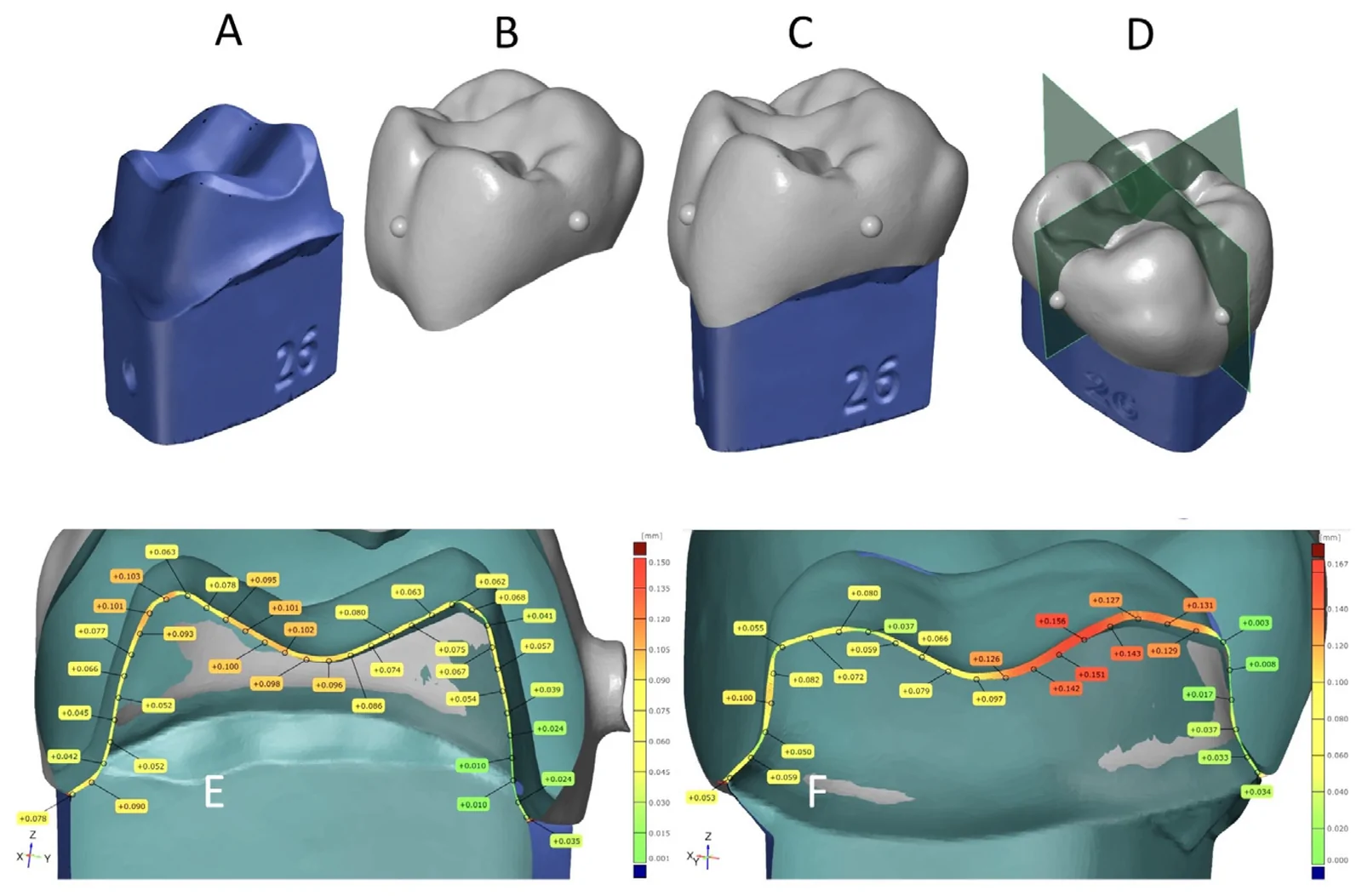
Evaluation of the internal and marginal adaptation of provisional crowns using the “triple-scan” method: scanning of the tooth preparation (**A**); scanning of the provisional crown (**B**); visualization of the provisional crown digitally seated on the preparation (**C**); implementation of buccopalatal and mesiodistal cross-sections of the seated crown based on surface reference spheres (**D**); and buccopalatal (**E**) and mesiodistal (**F**) evaluative cross-sections.

Internal adaptation was assessed along the internal aspects of the sections, whereas marginal adaptation was measured at the two most cervical points of each sectioning plane, corresponding to the buccal, palatal, mesial, and distal margins. Consequently, four marginal adaptation points were recorded per crown (Figure 6).

Statistical analysis was conducted using R software (version 4.3.2; R Core Team, Vienna, Austria, 2024). The sample size calculation was performed a priori using G Power software (version 3.1.9.7; Heinrich Heine Universität, Düsseldorf, Germany), utilizing a one-way ANOVA for fixed effects, with an effect size of f = 0.5 (large), a significance level of 5% (α = 0.05), and a statistical power of 95% (1–β = 0.95). The calculation was based on the comparison among the five build orientations within each 3D-printing system and resulted in a minimum required sample size of 16 specimens per group.

Considering the adopted experimental design, the study comprised 18 independent groups, including three milling groups and fifteen 3D-printing groups—the latter resulting from the combination of three technologies (LCD, DLP, and PolyJet) and five printing orientations per technology (0°, 30°, 45°, 60°, and 90°). For each group, 20 provisional crowns were fabricated, totaling 360 specimens. The sample size of 20 specimens per group exceeded the minimum requirement established by the a priori calculation and was consistently adopted across all experimental groups.

Descriptive statistics were calculated for each experimental group, including mean and standard deviation, to characterize the data distribution for each evaluated angle and equipment. Data normality was verified using the Shapiro–Wilk test, and homogeneity of variances was confirmed by the Levene test. As the data followed an approximately normal distribution with homogeneous variances, mean comparison tests were performed via one-way analysis of variance (ANOVA), applied separately to each dependent variable (marginal adaptation, internal adaptation, and internal surface accuracy).

For each measurement, significance tests were applied exclusively to the mean values of each group. Standard deviations were analyzed as a complementary parameter to interpret data variability within each experimental condition.

Statistically significant differences between groups were identified using the Tukey post hoc test, with a significance level of 5% (α = 0.05). Groups with distinct letters were considered significantly different according to the results of this test. All analyses were conducted using R software (version 4.3.2; R Core Team, Vienna, Austria, 2024).

Initially, a comparison was made between the different printing orientations evaluated for each printer, individually considering the parameters of marginal adaptation, internal adaptation, and internal surface accuracy. For each device, the best-performing orientation was selected as the angle exhibiting the lowest mean value in the greatest number of the three evaluated outcomes. The results obtained at this specific angle were considered representative of the respective printer and were used in subsequent comparisons across all evaluated technologies.

## 3. Results

The sample size was determined a priori using G*Power software v3.1.9 (f = 0.5, α = 0.05, power = 0.95) based on the comparison among the five build orientations within each 3D-printing system, resulting in a minimum requirement of 16 specimens per group. A sample size of 20 specimens per group was adopted consistently across all experimental groups, resulting in 360 specimens distributed across 18 independent groups (3 milling and 15 3D-printing groups). Data normality and homogeneity of variances were confirmed using the Shapiro–Wilk and Levene tests (*p* > 0.05), respectively, supporting the use of one-way ANOVA (Table 1) followed by Tukey’s post hoc test for multiple comparisons.

### 3.1. Impact of Build Orientation on 3D Printed Crowns

The build angle significantly influenced all evaluated parameters across the additive technologies (*p* < 0.05).

- Optimal Angles: 0° and 30° orientations consistently demonstrated the highest fidelity. For the Stratasys Objet30 (PolyJet) system at 0°, the marginal discrepancy was 0.040/pm 0.017/mm and the intaglio surface deviation (RMS) was 0.090/pm 0.015/mm.
- Steeper Angles: Performance deteriorated significantly at 60° and 90°. In the PolyJet system, the 90° orientation resulted in a marginal discrepancy of 0.140/pm 0.056/mm and an intaglio deviation of 0.137/pm; 0.021/mm.

### 3.2. Comparative Analysis of Manufacturing Systems

**Figure 7:**
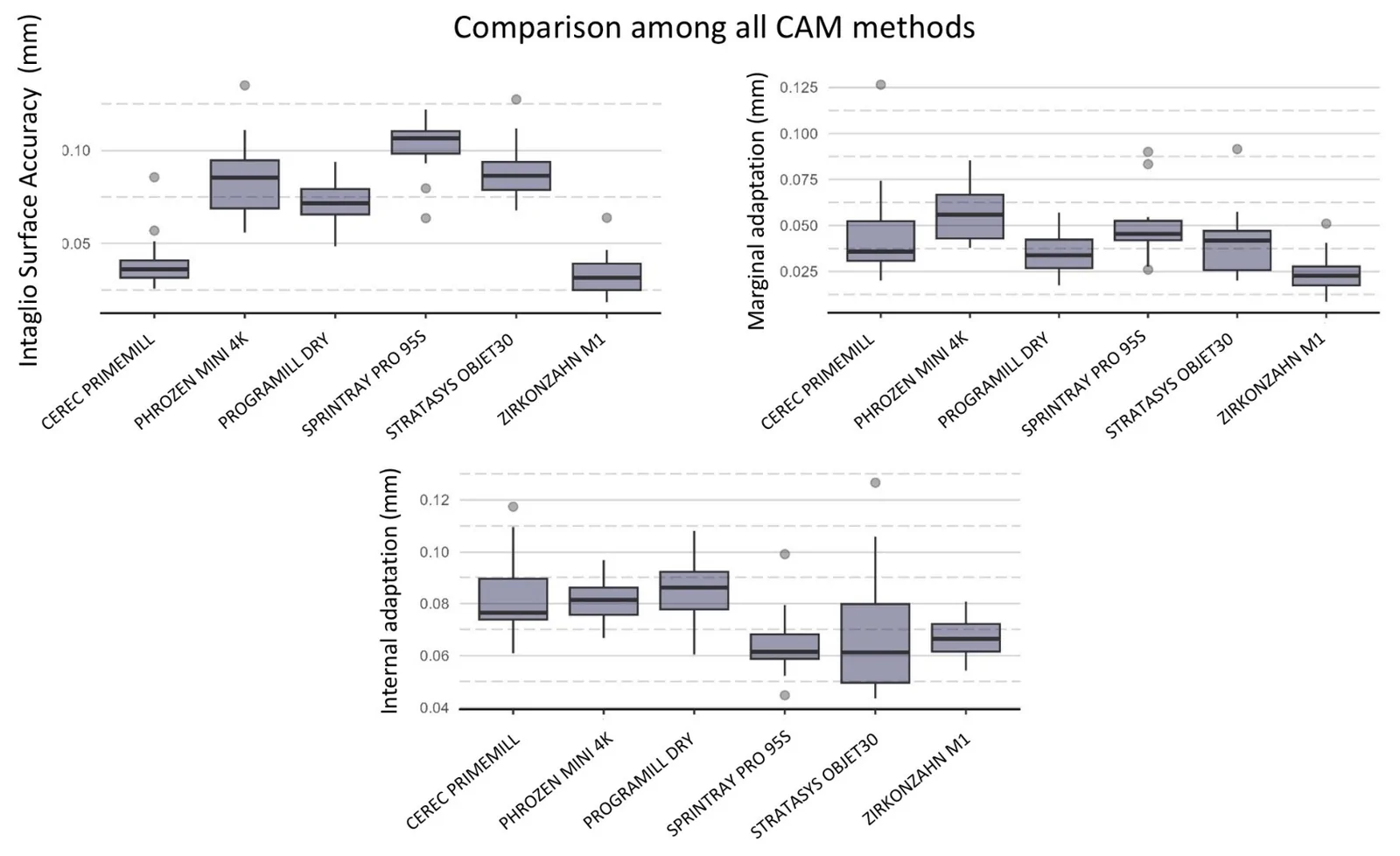
Comparison of all milling units and 3D printers using an established optimized 3D-printing angle. Circles represent outliers beyond 1.5 times the interquartile range (IQR) and do not indicate statistical differences. Statistical comparisons were performed using one-way ANOVA followed by Tukey’s post hoc test, with the significance level set at α = 0.05.

For the final comparison, the best-performing orientation for each printer was used (Table 2) and the results for all manufacturing systems are presented in Figure 7. In the evaluation of internal surface accuracy, the lowest mean RMS values were observed for the Zirkonzahn M1 (0.033 mm) and CEREC Primemill (0.039 mm) milling units; both were classified in group “a,” demonstrating significantly superior trueness compared to all other methods. The PrograMill milling unit presented an intermediate value (0.073 mm) and was assigned to group “b.” Among the 3D printers, the Phrozen (0.084 mm) was classified in group “bc,” the Stratasys (0.090 mm) in group “c,” and the SprintRay (0.103 mm) in group “d,” showing a statistically significant difference relative to all other technologies. For all six groups, the ANOVA test results for intaglio surface accuracy were: df = 5, 114; F = 80.15; *p* < 0.001; partial η^2^ = 0.779.

Regarding precision—represented by the standard deviation of the RMS values—most evaluated methods exhibited low internal variability. The lowest standard deviations were observed for the Zirkonzahn M1 (±0.011 mm), PrograMill (±0.011 mm), CEREC Primemill (±0.014 mm), and SprintRay (±0.014 mm) technologies, indicating high consistency of results within each group. The Stratasys (±0.015 mm) and Phrozen (±0.020 mm) printers showed slightly higher standard deviations, suggesting greater dispersion among samples under these conditions.

In the assessment of marginal adaptation, the Zirkonzahn M1 milling unit exhibited the lowest mean value (0.025 mm) and was classified in group “a.” The PrograMill obtained a mean of 0.035 mm and was allocated to group “ab,” showing no statistical difference compared to the Zirkonzahn. The Stratasys presented a mean of 0.040 mm but was grouped under the letter “b,” indicating performance statistically superior to that of the Phrozen (0.057 mm), which was classified in group “c.” The CEREC Primemill (0.044 mm) and the SprintRay (0.048 mm) were both classified in group “bc.” For all six groups, the ANOVA test results for marginal adaptation were: df = 5, 114; F = 9.19; *p* < 0.001; η^2^_p_ = 0.287. Regarding precision, all methods exhibited low variability, with standard deviations ranging between ±0.011 mm (PrograMill) and ±0.024 mm (CEREC Primemill).

In the evaluation of internal adaptation, the SprintRay printer presented the lowest mean value (0.064 mm) and was classified in group “a.” The Stratasys and the Zirkonzahn M1 milling unit were also categorized in group “a,” with means of 0.068 mm and 0.067 mm, respectively, showing no statistical differences compared to the SprintRay. Group “b” included the CEREC Primemill (0.082 mm), PrograMill (0.085 mm), and Phrozen (0.081 mm), which demonstrated statistically inferior but equivalent performance to one another. For all six groups, the ANOVA test results for internal adaptation were: df = 5, 114; F = 8.34; *p* < 0.001; η^2^_p_ = 0.268. In terms of precision, all technologies exhibited standard deviations of up to ±0.015 mm, with the exception of the Stratasys and CEREC Primemill, which displayed higher variability (±0.023 mm).

## 4. Discussion

The present study demonstrated that build orientation significantly influences the quality of 3D-printed provisional crowns across all additive technologies investigated. The findings indicated that lower angles, specifically 0° and 30°, yielded the highest intaglio surface accuracy and superior internal and marginal fit, consistently outperforming steeper orientations such as 90°. These results align with research by Ryu et al. [24], who observed that increased build angles lead to greater dimensional deviations due to the “staircase effect” and the increased need for support structures on critical internal surfaces. Furthermore, the stability provided by lower angulations during the layer-by-layer photopolymerization process minimizes the risk of geometric distortion [25]. Our data confirm that while 3D printing offers high flexibility, strict adherence to optimized orientation protocols is essential to maintain clinical acceptability, particularly for complex molar geometries [26,27].

When comparing manufacturing methods, subtractive technologies (milling) demonstrated overall superiority in intaglio surface accuracy and marginal fit compared to additive methods. Among the milling systems, performance varied according to the evaluated outcome, with Zirkonzahn M1 showing particularly high geometric fidelity and low variability across specimens. These findings suggest that milling performance cannot be attributed solely to the number of machining axes. This superior performance is likely due to the use of industrially pre-polymerized PMMA blocks, which offer high dimensional stability and a high degree of monomer conversion compared to the liquid resins used in 3D printing, which are susceptible to polymerization shrinkage [11,17]. These findings are consistent with previous literature suggesting that milling remains the “gold standard” for marginal adaptation, as the mechanical precision of 5-axis units allows for more complex and accurate tool paths in the cervical region [14,15].

Interestingly, no statistically significant differences were found between additive and subtractive methods regarding internal fit. While milling was more accurate in reproducing the overall internal geometry (intaglio accuracy), both methods successfully achieved the programmed cement space (80 μm) within clinically acceptable limits. This suggests that modern 3D printers, when properly calibrated, can rival milling machines in providing adequate space for the luting agent, ensuring a passive fit [28,29]. Similar observations were made by Wu et al. [30] and Earar et al. [31], who reported that while marginal discrepancies vary between methods, the internal adaptation of both printed and milled crowns often falls within the same clinical range. This parity indicates that for internal adaptation alone, 3D printing is a highly viable alternative to traditional milling.

The performance among the three 3D printing technologies also revealed distinct strengths: LCD (Phrozen Mini 4K) achieved the best intaglio surface accuracy, DLP (SprintRay Pro95S) showed superior internal fit, and PolyJet (Stratasys Objet30) provided the best marginal adaptation. The LCD and DLP systems processed the same resin (VarseoSmile Temp) yet differed significantly in intaglio surface accuracy (0.084 mm versus 0.103 mm) and marginal adaptation (0.057 mm versus 0.048 mm), which indicates that, for a given material, light source characteristics, pixel resolution, and post-processing routines exert a greater influence on accuracy than the resin itself [12,23,31,32,33]. The high accuracy of the LCD system can be attributed to its fine pixel resolution and manual control over exposure parameters, as noted in studies by various authors [20,21]. Conversely, the PolyJet technology’s superiority in marginal fit may stem from its unique droplet-on-demand mechanism, which eliminates the tray-peeling forces common in VAT polymerization (LCD/DLP) that can distort thin marginal edges [34]. These technological nuances highlight that the “best” printer may depend on the specific clinical priority, whether it be geometric fidelity or marginal sealing [26,28]. Within the subtractive group, the three units processed PMMA blanks from different manufacturers, and differences in filler content, molecular weight, and machinability among these blanks may have contributed to the intermediate intaglio accuracy of one of the milling systems [35], alongside the differences in bur geometry and milling strategy [14,15].

Despite the rigorous methodology, this study has several limitations that should be acknowledged. As an in vitro investigation, it utilized a standardized PMMA preparation of a single tooth type (maxillary first molar), which may not fully represent the complexities of various clinical preparations or the challenges of multi-unit bridges. Moreover, the controlled experimental setting cannot fully reproduce the dynamic intraoral environment, where exposure to oral fluids, interactions with surrounding soft tissues, and variability inherent to clinical manipulation may influence restoration seating and adaptation. These factors should be considered when extrapolating the present findings to clinical performance.

Another drawback of the study is that the final comparison between additive and subtractive methods used the best-performing build angle for each 3D-printing system, whereas the milling systems were evaluated under a single manufacturing condition. Thus, this comparison reflects the best-performing condition for each additive technology rather than a like-for-like comparison under equivalent manufacturing conditions.

The absence of a cementation phase is another significant limitation, as the hydraulic pressure and film thickness of the cement can alter the final seating and adaptation of the crowns in a clinical setting [30]. Additionally, the study focused on specific resin and block materials; different proprietary formulations might yield different results regarding shrinkage and stability. Finally, the use of a single high-end intraoral scanner (Primescan) for the triple-scan evaluation, while accurate, may not reflect the results obtained with more common or lower-resolution scanning systems [36]. Further, potential errors inherent to the digital alignment procedures may have influenced the measurements.

Future investigations should extend the assessment of manufacturing performance beyond the initial accuracy and fit of provisional restorations. Evaluating how these restorations behave under conditions that reproduce functional and environmental challenges may provide a more comprehensive understanding of their clinical performance. Long-term changes in dimensional stability, mechanical behavior, and adaptation could also reveal whether the differences observed among manufacturing methods persist over time and have an impact on restoration longevity.

## 5. Conclusions

Both subtractive and additive CAD/CAM methods are capable of producing provisional crowns that meet clinical requirements for accuracy and fit. However, milling systems offer greater dimensional predictability and superior marginal adaptation compared to 3D printing. For 3D printing, the build orientation is a critical success factor, with 0° and 30° angles recommended to minimize geometric errors. While 3D printing is a cost-effective and flexible alternative, milling currently remains the more precise method for ensuring the highest level of intaglio fidelity and marginal seal in provisional prosthodontics. Nevertheless, the relatively small differences observed between methods may not be clinically meaningful, particularly when values remain within accepted clinical thresholds.

## Figures and Tables

**Table 1:** Comparison of the six systems. df = degrees of freedom (between, within); η^2^_p_ = partial eta squared. Printed groups are represented by their optimal build orientation (30° for Phrozen Mini 4K and SprintRay Pro95S; 0° for Stratasys Objet30). n = 20 per group.

Dependent Variable	df	F	*p*	η^2^_p_
Marginal adaptation	5, 114	9.19	2.28 × 10^−7^	0.287
Internal adaptation	5, 114	8.34	9.56 × 10^−7^	0.268
Intaglio surface accuracy	5, 114	80.15	1.12 × 10^−35^	0.779

**Table 2:** Mean and standard deviation values for all CAM methods tested in the study. For each additive manufacturing method (Phrozen, SprintRay, and Stratasys), values correspond to the best-performing build angle among the five angles tested. Within each column, groups sharing at least one superscript letter did not differ significantly (Tukey’s HSD, α = 0.05); “A” indicates the lowest discrepancy values and thus the most favorable performance.

CAM Method	Marginal Adaptation (mm)	Internal Adaptation (mm)	Intaglio Surface Accuracy (mm)
Phrozen Mini 4k	0.057 ± 0.016 ^C^	0.081 ± 0.009 ^B^	0.084 ± 0.020 ^BC^
SprintRay PRO 95S	0.048 ± 0.015 ^BC^	0.064 ± 0.012 ^A^	0.103 ± 0.014 ^D^
Stratasys Objet30	0.040 ± 0.017 ^B^	0.068 ± 0.023 ^A^	0.090 ± 0.015 ^C^
CEREC Primemill	0.044 ± 0.024 ^BC^	0.082 ± 0.015 ^B^	0.039 ± 0.014 ^A^
PrograMill Dry	0.035 ± 0.011 ^AB^	0.085 ± 0.011 ^B^	0.073 ± 0.011 ^B^
Zirkonzahn M1	0.025 ± 0.011 ^A^	0.067 ± 0.007 ^A^	0.033 ± 0.011 ^A^

## Data Availability

The original contributions presented in this study are included in the article. Further inquiries can be directed to the corresponding author.

## Abbreviations

The following abbreviations are used in this manuscript:

STL	Standard Tessellation Language
3D	Three-dimensional
LCD	Liquid Crystal Display
DLP	Digital Light Processing
